# Prioritizing candidate genes for fertility in dairy cows using gene-based analysis, functional annotation and differential gene expression

**DOI:** 10.1186/s12864-019-5638-9

**Published:** 2019-03-29

**Authors:** Zexi Cai, Bernt Guldbrandtsen, Mogens Sandø Lund, Goutam Sahana

**Affiliations:** Center for Quantitative Genetics and Genomics, Department of Molecular Biology and Genetics, Aarhus University, 8830 Tjele, Denmark

**Keywords:** Dairy cattle, Female fertility, Gene-base analysis, Gene annotation, RNA-seq

## Abstract

**Background:**

An unfavorable genetic correlation between milk production and fertility makes simultaneous improvement of milk production and fertility difficult in cattle breeding. Rapid genetic improvement in milk production traits in dairy cattle has been accompanied by decline in cow fertility. The genetic basis of this correlation remains poorly understood. Expanded reference populations and large sets of sequenced animals make genome-wide association studies (GWAS) with imputed markers possible for large populations and thereby studying genetic architecture of complex traits.

**Results:**

In this study, we associated 15,551,021 SNPs with female fertility index in 5038 Nordic Holstein cattle. We have identified seven quantitative trait loci (QTL) on six chromosomes in cattle. Along with nearest genes to GWAS hits, we used gene-based analysis and spread of linkage disequilibrium (LD) information to generate a list of potential candidate genes affecting fertility in cattle. Subsequently, we used prior knowledge on gene related to fertility from Gene Ontology terms, Kyoto Encyclopedia of Genes and Genomes pathway analysis, mammalian phenotype database, and public available RNA-seq data to refine the list of candidate genes for fertility. We used variant annotations to investigate candidate mutations within the prioritized candidate genes. Using multiple source of information, we proposed candidate genes with biological relevance underlying each of these seven QTL. On chromosome 1, we have identified ten candidate genes for two QTL. For the rest of chromosomes, we proposed one candidate gene for each QTL. In the candidate genes list, differentially expressed genes from different studies support *FRAS1, ITGB5, ADCY5, and SEMA5B* as candidate genes for cow fertility.

**Conclusion:**

The GWAS result not only confirmed previously mapped QTL, but also made new findings. Our findings contributes towards dissecting the genetics for female fertility in cattle. Moreover, this study shows the usefulness of adding independent information to pick candidate genes during post-GWAS analysis.

## Background

Dairy cattle breeding has achieved large increases in milk production traits; however, simultaneously cow fertility has declined [1]. A negative genetic correlation exists between yield and cow fertility [2]. This negative genetic correlation of milk yield and its compositions with fertility is assumed to be due to the negative energy balance of high-producing cows during the peak of lactation [2]. Good fertility is essential for the overall economy of dairy farming [3]. Multiple measures of fertility such as timing of estrus, pregnancy rate, days open, and services per conception have been devised [4].

Numerous biological pathways are involved in processes related to cow fertility. Thus fertility is a complex trait affected by many genes or variants, each typically with small effects [5]. This means that power of detection of individual genetic variants typically will be low. With next-generation-sequencing (NGS) combined with imputation, we are able to map variants at the whole-genome-sequencing (WGS) level in large populations. For cattle, the availability of data from 1000 Bull Genome Project further facilitates the use of WGS for GWAS [6]. The sequence data is expected to include most causal variants affecting a polygenic traits or at least markers in strong LD with causal variants.

Several GWAS and QTL mapping studies for fertility in different cattle populations have been conducted. Two QTL, one at 26 cM of chromosome 3 and one at 107 cM of chromosome 7 were reported in French Holstein cattle [7]. In another study for French Holstein cattle, the position of QTL on chromosome 3 was at 19 cM [8]. In US Holstein, Ashwell et al. [9] reported one strong signal on chromosome 18 affecting pregnancy rate, along with other QTL on chromosome 6, 14, 16, 27, and 28 [9]. In Nordic Red breeds, Kadri et al. [10] identified the causative mutation associated with a major fertility QTL located on chromosome 12 was a 660-Kb deletion encompassing four genes [10]. In the Canadian Holstein population, there was fertility QTL on chromosome 21 located between 53 to 59 Mb. Several QTL for fertility were reported in the Nordic Holstein cattle population located on chromosomes 1, 4, 7, 9, 11, and 13 [11]. Moreover, some of these QTL were also found to be segregating in Nordic Red cattle and Danish Jersey [11]. Nonetheless, overlap of QTL location among different populations is poor [11]. This inconsistency illustrates the challenges in finding candidate genes and mapping causal variants for cow fertility. However, more and more available information about function of genes and their annotation in human and other model species can shed light on genes affecting cow fertility. The integration of additional sources of functional information helps to identify candidate genes for fertility to the benefit of research and breeding of dairy cattle.

The objectives of our study were: 1) using WGS markers for GWAS to find associations between markers and cow fertility; 2) using gene-based association statistics, Gene Ontology (GO) terms, Kyoto Encyclopedia of Genes and Genomes (KEGG) pathway analysis, mammalian phenotype database (MPD), along with publicly available differential expression gene (DEG) to prioritize candidate genes.

## Results

### Association analysis for fertility

**Fig. 1:**
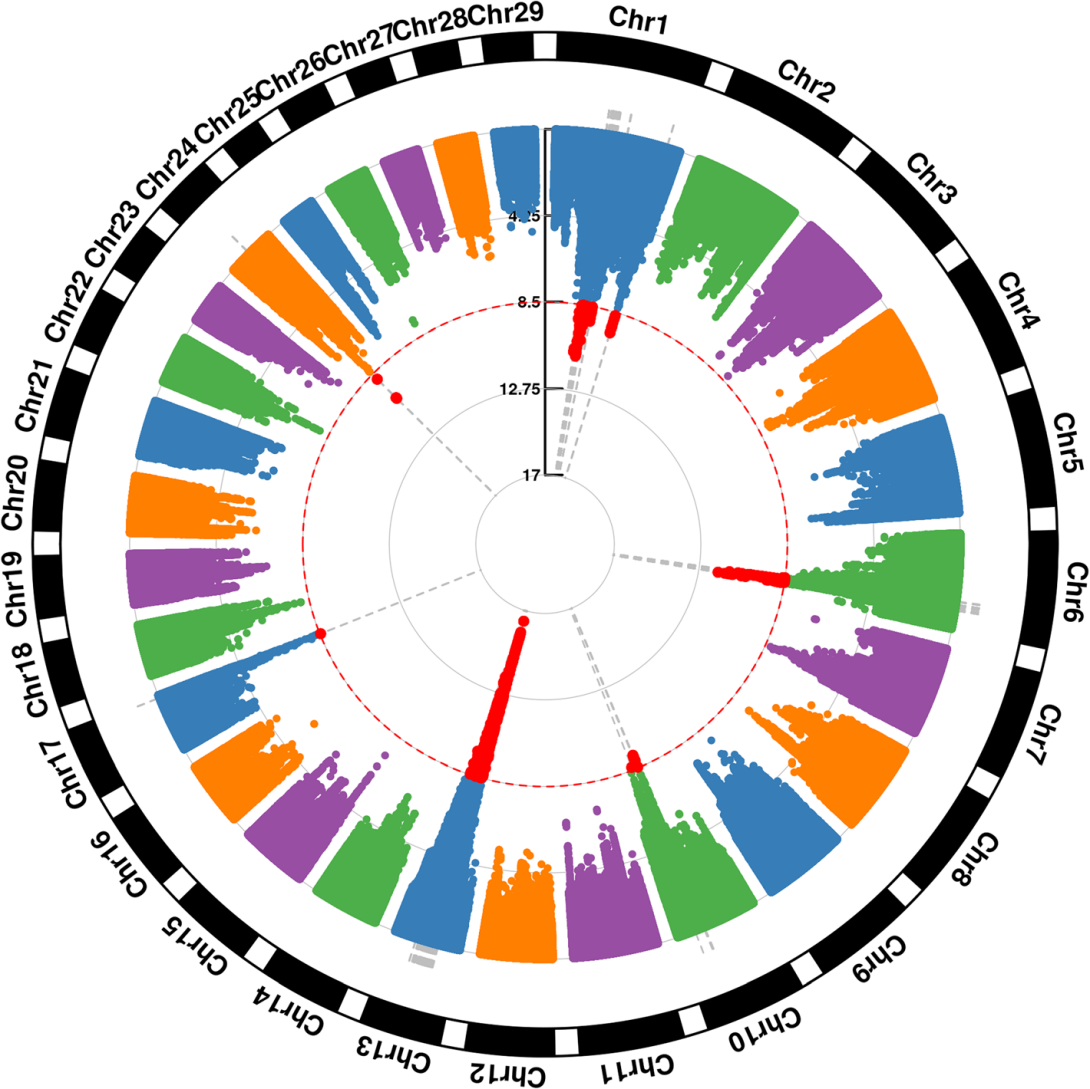
Circular Manhattan plot for association of SNP with fertility index in Nordic Holstein cattle. The dashed red line indicates the genome-wide significance level [−log_10_(P) = 8.5]. Red dots indicate genome-wide significantly associated SNP

Using a previously published approach [12], we performed GWAS and found seven independent QTL (−log_10_(P) > 8.5) across *Bos taurus* autosomes (BTA) 1, 6, 10, 13, 17 and 24 (Table 1 and Fig. 1). In total, 5806 SNPs exceeded the genome-wide significance level. The most significant signal was on BTA13 with the lead SNP located at 32,852,133 bp (rs210238678). This QTL was close to previous reported QTL in Nordic Holstein cattle (at BTA13: 33,903,159 bp) [13]. The second strongest association signal was on BTA6 (95,867,927 bp, rs41567777) with *GK2* as the closest gene. *GK2* belongs to GO terms “carbohydrate metabolic process” and “galactose metabolic process”. On chromosome 1, we identified two QTL. The first had its lead SNP at 69,742,415 bp (rs208311936) which is an intronic variant in the *UMPS* gene. The second had its lead SNP at 140,785,028 bp (rs385628476), an intronic variant in the *BRWD1* gene. *UMPS* belong to GO terms “female pregnancy” (The set of physiological processes that allow an embryo or foetus to develop within the body of a female animal that covers the time from fertilization of a female ovum by a male spermatozoon until birth) which make it a good candidate for fertility. For *BRWD1*, there is no documented biological connection between the available functional annotations with the gene and fertility. On BTA 24, the lead SNP was located at 29,556,826 bp (rs380439408). The closest gene is a novel gene, *ENSBTAG00000044212.* On BTA10, we identified SNP at 68,534,665 bp (rs211204488) as the lead SNP. The annotated element closest to this lead SNP is U7 snRNA and *SLC39A12* is the closest gene. The lead SNP on BTA17 was located at 71,393,345 bp (rs110812733). This lead SNP is located close to *LIF* gene which is involved in many biological processes including “maternal process involved in female pregnancy”, thereby making it a good candidate gene for female fertility.

**Table 1:** Lead SNPs from genome-wide associated regions for female fertility in Nordic Holstein cattle

BTA	BP	effect	-log_10_(P)	Interval	Gene (distance in bp)	Annotation
1	69,742,415	3.68	11.03	68,745,058~69,992,433	*UMPS*	intron
1^a^	140,785,028	1.91	9.53	139,838,183~141,035,632	*BRWD1*	intron
6	95,867,927	2.15	11.80	95,377,650~96,118,034	*GK2* (14,991)	intergenic
10	68,534,665	2.08	9.16	67,917,166~68,784,933	U7 snRNA (29,099)	intergenic
13	32,852,133	−3.09	16.48	31,866,274~33,102,422	*SLC39A12* (100,247)	intergenic
17	71,393,345	1.83	8.54	70,501,607~71,656,270	*LIF* (20,510)	intergenic
24	29,556,826	2.32	10.18	28,794,256~29,807,379	*ENSBTAG00000044212* (65,604)	intergenic

Base positions are given as positions in UMD 3.1.1 [49]. Lead SNPs identified in the second round are marked by^a^. The method to define QTL intervals was described in Methods

#### Gene-based analysis within a QTL interval

Due to small effective population sizes of major dairy breeds, the LD extends over long distances [14]. We used a linear-regression model implemented in MAGMA [15], which considers LD, to perform gene-based association analysis within the QTL intervals. It resulted in 447 genes being genome-wide significant (*P* < 2.46 × 10^− 6^) for seven QTL. To refine this gene list, we extracted all significant SNP with LD with the lead SNP (r^2^ > 0.2). The genes significant in gene-based analysis closest to these SNPs were identified (Table 2). Some of the candidate genes identified based on proximity to the lead SNP were also in this list. However, the candidate genes suggested by gene-based analysis for BTA10:68534665 (rs211204488), BTA13:32852133 (rs210238678), and BTA24:29556826 (rs380439408) did not include the genes closest to the lead SNP.

**Table 2:** Candidate genes identified by gene-based analysis

QTL	Official Gene name	Location	-log_10_(P)
1: 69742415	*DIRC2*	67,722,242–67,810,789	56.38
	*SEMA5B*	67,840,125–67,878,028	50.97
	*PDIA5*	68,012,078–68,101,852	47.22
	*SEC22A*	68,170,572–68,232,640	23.42
	*ADCY5*	68,263,141–68,420,954	61.35
	*MYLK*	68,580,144–68,667,822	47.83
	*ROPN1*	68,954,525–68,989,623	22.27
	*KALRN*	69,248,613–69,724,961	62.48
	*UMPS*	69,732,778–69,782,823	31.65
	*ITGB5*	69,801,844–69,899,676	40.59
	*HEG1*	70,027,330–70,106,415	44.68
	*SLC12A8*	70,126,992–70,250,895	30.26
	*UBXN7*	71,542,376–71,573,340	33.56
	*RNF168*	71,629,579–71,653,254	45.44
1: 140785028	*PSMG1*	140,726,628–140,739,236	27.92
	*BRWD1*	140,741,647–140,863,405	22.38
6: 95867927	*FRAS1*	94,711,652–95,055,569	33.89
	*PAQR3*	95,409,078–95,441,932	22.86
	*GK2*	95,882,918–95,884,576	13.76
	*ENSBTAG00000032034*	95,908,624–95,908,893	11.44
10: 68534665	*KTN1*	68,238,010–68,351,879	42.59
	*OTX2*	69,458,818–69,462,865	13.60
13: 32852133	*KLF6*	44,945,068–44,952,151	17.28
17: 71393345	*LIF*	71,413,855–71,418,166	14.25
24: 29556826	*SPIRE1*	43,323,645–43,488,851	12.47

#### Post GWAS prioritization of candidate genes using multiple sources of information

To prioritize candidate genes obtained from nearest to lead SNP and gene-based analysis, we used GO, KEGG and MPD [16] as additional biological support to candidate genes. In addition to *UMPS* and *LIF* that already had known biological connection to fertility, the following genes also have at least one source of information to support them as candidate genes. *KALRN* belongs to the GO terms “maternal process involved in parturition”. *HEG1* belongs to the GO term “post-embryonic development”. *FRAS1* belongs to the GO term “embryonic limb morphogenesis”. *OTX2* belongs to the GO term “positive regulation of embryonic development”. *SPIRE1* belongs to the GO term “establishment of meiotic spindle localization”. *ADCY5* belongs to KEGG pathway “oocyte meiosis”. *SPIRE1* belongs to the KEGG pathway “dorso-ventral axis formation”. *MYLK* belongs to the KEGG pathway “oxytocin signaling pathway” which is involved in stimulation of uterine contractions during parturition. By searching MPD [16], we found that mutations in *BRWD1* of mouse may cause female infertility. Mutations in *HEG1* of mouse may cause partial embryonic lethality between embryo turning and the completion of organogenesis. Mutations in *KLF6* may cause abnormal embryonic hematopoiesis in mouse. Mutations in *LIF* may cause failure of embryo implantation in mouse. Mutations in *OTX2* may cause reduced fertility and embryonic growth arrest. Mutation in *PSMG1* may cause abnormal embryo development in mouse.

#### Validation of candidate genes from differential expressed genes

To validate our list of candidate genes and give biological evidence to candidate genes, we retrieved the list of DEGs from previous studies. We chose three RNA-seq datasets with a case-control design. The first dataset [17] contains DEGs in the endometrium and the corpus luteum of Holstein cows selected for high and low fertility. No overlap between our candidate genes and the DEGs list of this dataset was observed. The second dataset contains DEGs in uterine biopsies from pregnant and non-pregnant cows [18]. *FRAS1* is the only one candidate gene appearing in our list and the second dataset. The third dataset contains DEGs from the endometrium or conceptuses in cows classified as having high fertility, lower fertility, or as infertile [19]. Twelve of our candidate genes including *ITGB5*, *ADCY5,* and *SEMA5B* also appeared in the third dataset (Table 3). Moreover, the change in expression of these three genes was in the same direction. In addition, *FRAS1* from two different studies and tissues showed same direction of response to pregnancy.

**Table 3:** Overlap gene between candidate genes and DEGs from previous study

Gene name	tissue	design	DE direction
*FRAS1*	uterine biopsies	pregnant vs non-pregnant animals [18]	Down
	endometrium	pregnant vs non-pregnant high-fertile animals [19]	Down
*ITGB5*	endometrium	pregnant vs non-pregnant high-fertile animals [19]	Up
	endometrium	pregnant vs non-pregnant subfertile animals [19]	Up
*KLF6*	endometrium	pregnant vs non-pregnant high-fertile animals [19]	Up
*MYLK*	endometrium	pregnant vs non-pregnant high-fertile animals [19]	Down
*DIRC2*	endometrium	pregnant vs non-pregnant high-fertile animals [19]	Up
*KALRN*	endometrium	pregnant vs non-pregnant high-fertile animals [19]	Down
*ADCY5*	endometrium	pregnant vs non-pregnant high-fertile animals [19]	Down
	endometrium	pregnant vs non-pregnant high-fertile animals [19]	Down
*SEMA5B*	endometrium	pregnant vs non-pregnant subfertile animals [19]	Down
	endometrium	pregnant vs non-pregnant high-fertile animals [19]	Down
*OTX2*	conceptuses	subfertile vs high-fertile animals [19]	Up

Note, high-fertile (100% pregnancy rate) and subfertile (25 to 33% pregnancy rate) using serial transfer (*n* = 3 to 4 rounds) of a single in vitro-produced embryo on day 7 followed by pregnancy determination on day 28 [19]

#### Variants annotation of the variants in LD with lead SNP

**Fig. 2:**
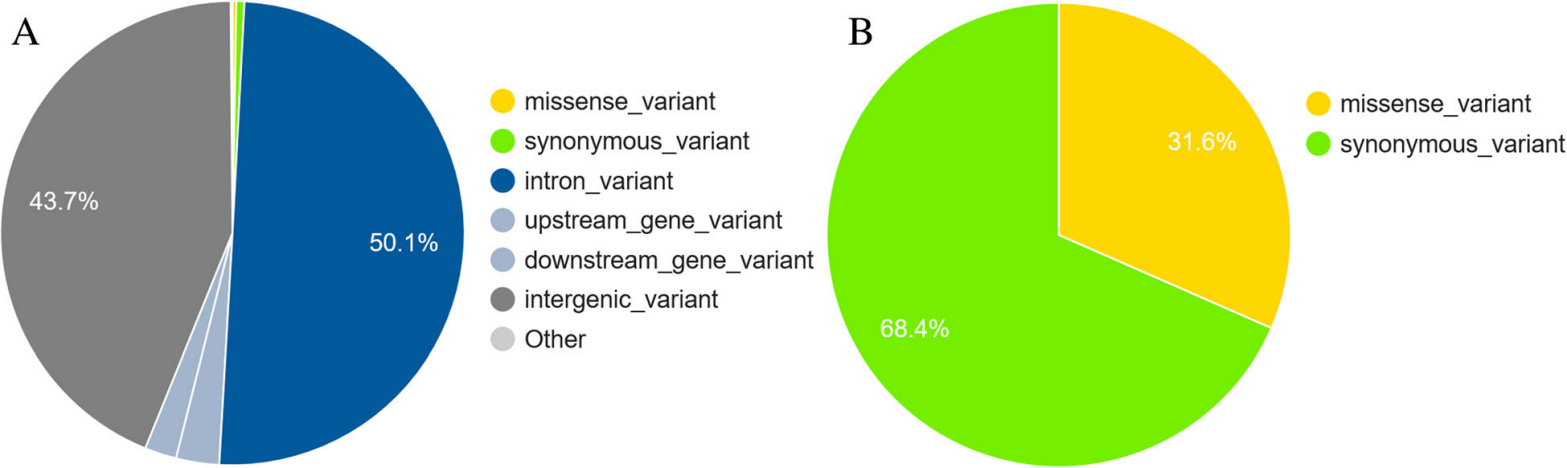
The VEP annotation of SNPs in linkage disequilibrium (r^2^ > 0.20) with lead SNPs. **a**) Summary of annotations of all imputed variants. **b**) Summary of annotations of variants which change the protein coding sequence

Lead SNP may or may not be the causal variants [12]. To refine our search, we annotated all significant SNP in LD with the lead SNP (r^2^ > 0.2). This included 2283 variants. Half of these variants (1153, Fig. 2A) were intron variants, followed by intergenic variants (1007, Fig. 2A). Among variants located in coding sequences, most (13, Fig. 2B) were synonymous variants, followed by missense variants (6, Fig. 2B). Some variants had predicted functional consequences: *KALRN* contained a splice acceptor variant (rs109819533, BTA1:69652189). Two missense variants (BTA13:32679690, rs464438710 and BTA1:69673871, rs209885271) were reported as “deleterious” by SIFT [20]. They were located in the genes *SLC39A12* and *KALRN*.

#### Candidate genes for fertility

In summary, we proposed candidate genes for all seven QTL associated with fertility in Nordic Holstein cattle (Table 4). For BTA1: 69742415 (rs208311936), we found several candidate genes with different supporting evidence. In this list, *KALRN* has two potential causal mutations (rs109819533, BTA1:69652189 and BTA1:69673871, rs209885271). For the second lead SNP at BTA1:140785028 (rs385628476), we identified two candidate genes. Both of them have evidence from MPD. For the rest of the QTL, we propose one candidate gene for each QTL.

**Table 4:** Candidate genes for lead SNPs for each identified QTL affecting fertility with list of types of supporting evidence

Lead SNP BTA:Base position	rsID (dbSNP v. 150)	Office Gene name	Evidence
1: 69742415	rs208311936	*DIRC*	DE
		*SEMA5B*	DE
		*ADCY5*	KEGG and DE
		*MYLK*	KEGG and DE
		*KALRN*	GO and DE
		*UMPS*	GO
		*ITGB5*	DE
		*HEG1*	GO and MPD
1: 140785028	rs385628476	*PSMG1*	MPD
		*BRWD1*	MPD
6: 95867927	rs41567777	*FRAS1*	GO,DE
10: 68534665	rs211204488	*OTX2*	GO,MPD and DE
13: 32852133	rs210238678	*KLF6*	DE
17: 71393345	rs110812733	*LIF*	GO, MPD
24: 29556826	rs380439408	*SPIRE1*	GO, KEGG

## Discussion

### Overlap of identified QTL in other cattle populations

Cow fertility in dairy cattle is a complex trait with many biological pathway involved and has a very low heritability. Non-genetic factors like body condition, farm management, feeding also affect fertility in cattle. Therefore, results show little agreement between studies [7, 10, 11, 13]. Besides, WGS has not been used extensively to study the genetic basis of variation in cow fertility in dairy cattle [21, 22]. The current study has identified seven QTL located on six different autosomes (Table 1). The QTL with its lead SNP at BTA17: 71,393,345 (rs110812733) was close to a QTL (70–73 Mb) for fertility reported in Brown Swiss cattle [21]. The lead SNPs for the QTL on BTA10, BTA13, and BTA24 were close to significant SNPs for female fertility traits previously reported in Danish and Swedish Holstein cattle [23]. The QTL on BTA1 and BTA6 are novel findings. Previous reported QTL were located outside QTL intervals obtained in the present study.

### Search for candidate genes

We proposed candidate genes for seven QTL identified in this study. In this list, some candidate genes are supported by literature reports of relevant biological functions related to fertility in other species. In mouse, it has been shown that *BRWD1* is essential for female fertility by epigenetic control of meiotic chromosome stability [24]. *KLF6* is a member of the Krüppel-like factor, and have a similar function to other Krüppel-like factors. These are indispensable for normal implantation [25]. Leukemia inhibitory factor (LIF) is a cytokine. It is required for blastocyst implantation in mice. Several studies have shown that *LIF* is important for the establishment of pregnancy [26]. Previous studies have shown that *SPIRE1* encodes a protein that drives two critical steps in asymmetric oocyte division in mice [27].

A similar strategy to prioritize candidate genes have helped identify several candidate genes for mastitis resistance [28]. In the present study, we faced two challenges like in our previous study [28]. The first challenge is choosing appropriate sources of annotations to prioritize candidate genes. Even though many biological processes are involved in fertility, we could only use the direct evidence that would affect conception and carrying the fetus since we cannot be sure what other pathway also involved. The second challenge is the long list of candidate genes provided by gene-based analysis. In the present study, we incorporated LD information in two ways: 1) using the *linreg* model from MAGMA [15]; 2) only considering genes close to significant SNPs in LD with lead SNP. We observed the cross-validation of DEGs from different studies or different animals in one study could provide better support. In this study, we utilized multiple RNA-seq datasets to validate our candidate gene list. One of the candidate genes, *FRAS1*, was found in two dataset with DEGs. In both datasets, differences in expression were in the same direction when comparing pregnant with non-pregnant animals. Three other candidate genes, *ITGB5*, *ADCY5*, and *SEMA5B* were DE in the third dataset in two different fertility-classified animals in the same directions when comparing pregnant with non-pregnant animals [19]. This indicated that, with the emergence of more functional study datasets permits the identification of better candidate genes with higher confidence based on biological support. Moreover, the successful combination of information from different sources to prioritize candidate genes suggested we would gain higher power with the improvement of the understanding of functional genomics.

### Limitation of the present study

The GWAS for cow fertility using imputed WGS marker could identify only a small number of QTL in our study. Both limited sample size and small contribution of individual QTL to the total phenotypic variance for fertility have contributed to low power of detection. Even though the genome-wide markers can explain a sizable proportion of the trait heritability, the genome-wide significant SNPs often explain only a small proportion of the trait heritability. Meta-analysis of GWAS summary statistics from several populations can improve the power [29] and map the QTL location precisely [29]. We used the fertility index as phenotype in our study. The breeding value for fertility index is calculated based on several component traits, which bring many biological processes together. GWAS for individual component traits of fertility index will help to interpret the results biologically. The imputed WGS marker set helped us to precisely identify the location of QTL intervals. The fertility index included in the Nordic breeding goal and the identified whole genome sequence variants, if included in the EuroGenomics custom SNP chip [30], can help in routine genomic prediction.

## Conclusions

In this study, we associated 15,551,021 imputed WGS SNPs in 5038 Nordic Holstein cattle with female fertility index in Nordic Holstein cattle. The GWAS helped us to find seven QTL across six cattle autosomes. We obtained potential candidate genes by 1) nearest genes; 2) gene-based statistical analysis plus LD information. Subsequently, we argued the potential candidate genes by GO, KEGG, MPD and multiple public available DEG dataset to propose candidate genes with biological support. Our finds extended our knowledge about female fertility in dairy cattle and showed the power of our strategy.

## Methods

### Phenotype and genotype data

Phenotypic records of fertility in this study were obtained from the Nordic Cattle Genetic Evaluation database (NAV, http://www.nordicebv.info/). The phenotype records used for association were de-regressed breeding values [31, 32] from the routine genetic evaluation by NAV and were available for 5038 progeny tested Holstein bulls. The fertility index includes breeding values for interval from first to last insemination (heifers and cows), interval from calving to first insemination (cows) and number of inseminations (heifers and cows).

We used two-step method previously described by Iso-Touru et al. [33] and Wu et al. [34] to impute WGS data. All bulls were genotyped with the Illumina BovineSNP50 BeadChip (54 k) ver. 1 or 2 (Illumina, San Diego, CA, USA). In the first step, we imputed 54 k genotypes to high-density (HD) by IMPUTE2 v2.3.1 [35]. The reference population for imputation included 1222 Holsteins, 1326 Nordic Red Dairy Cattle, and 835 Danish Jerseys genotyped by Illumina BovineHD BeadChip. In the second step, these imputed HD genotypes were imputed to WGS by Minimac2 [36] with a multi-breed reference of 1228 animals from *Run4* of the 1000 Bull Genomes Project [37] and additional data from Aarhus University (80 individuals, including 23 Holsteins, 30 Nordic Red Dairy Cattle, and 27 Danish Jersey) [38]. In this step of imputation, we performed in 5-Mb chunks with a buffer region of 0.25 Mb on either side. At the end, 22,751,039 bi-allelic variants were present in the imputed sequence data. After excluding SNPs with a minor allele frequency below 0.5% or with a large deviation from Hardy–Weinberg proportions (*P* < 1.0^− 6^), 15,551,021 SNPs on 29 autosomes in Nordic Holstein cattle were retained for association analyses. The detailed of this WGS dataset was published previously [34].

### Methodology of multiple QTL detection and estimation of genetic variants explained by QTL

We used our previous proposed method [12] to perform the association analysis. In brief, we literately ran GWAS using GCTA [39] for each chromosome by fitting the dose of lead SNP from previous literation as covariates. To reduce the false positive, we defined the lead SNP as the significant ones (experiment-wise 0.05 type I error rate after Bonferroni correction for 15,551,021 simultaneous tests corresponds to a threshold of –log_10_(P) ≈ 8.5) with the largest –log_10_(P) value in each literation and significant in first literation. To minimize the impact of random errors and imputation inaccuracy, we also checked whether the lead SNP is solo SNP. The solo SNPs are SNPs with no other significant SNP within a 1 Mb region. These SNPs were skipped in our analysis. The lead SNP in each round were collected to build a lead SNP list. The boundaries of each QTL region were defined as followed. If the SNP –log_10_(P) value of the flanking 1 Mb region around the lead SNP decreased by more than three units compared to the value of the lead SNP and the region was larger than 0.25 Mb, then we set this SNP as the boundary; otherwise, we set ±0.25 Mb from the lead SNP as the QTL boundary.

### LD calculation and variant annotation

The procedure for LD calculation and variant annotation were described in our previous study [28]. We used PLINK [40] to calculate the pairwise *r*^*2*^ between the lead SNP and all other SNPs on the same chromosome. All significant SNPs with *r*^*2*^ > 0.2 with the lead SNP were extracted for variants annotation. These SNPs were annotated by VEP (version 92) [41].

### Candidate genes identification and confirmation with RNA-seq data

We included nearest genes and gene-based analysis as a list of potential candidate genes list. For the nearest genes, we used bedtools [42] closest function to find the nearest genes (or function annotated feature) to the lead SNPs. For gene-based analysis, we used MAGMA [15]. In order to run MAGMA [15] for cattle, we should provide customer gene annotation file and reference population to MAGMA. For cattle genome annotation, we downloaded the gene information file from Ensembl gene build 92 [43] and converted them to MAGMA style location file. For reference population, 455 Holstein animals from the 1000 Bull Genome Project (*Run 6*) [37, 38] were used for this purpose. We performed MAGMA gene analysis with the GWAS result using the model *linreg.* The number of genes (including 5′- and 3′-UTRs) with at least one SNP was 20,356; thus, the *P*-value threshold for genome-wide significance for the gene-analysis was 2.46 × 10^− 6^. Significant SNPs in LD with lead SNPs were used to extract the closest or overlapping genes by bedtools [42] closest function from the list of significant genes from MAGMA [15]. These two sources of potential candidate genes were analyzed using DAVID [44] to retrieve the KEGG pathway annotations [45]. GO terms [46] associated with these genes were retrieved from Uniprot [47]. The MPD [16] was searched for mutations in these genes with known phenotypic effects related to fertility. At the meanwhile, the list of DEGs from three previous studies [17–19] were used to provide biological evidence for genes***.*** All the potential candidate genes with any biological support (GO, KEGG, MPD and DE) were proposed as final candidate genes.

## Abbreviations

BTA: *Bos taurus* autosome
DEG: differentially expressed gene
GO: Gene Ontology
GRM: genetic relationship matrix
GWAS: genome-wide association study
KEGG: Kyoto Encyclopedia of Genes and Genomes
LD: linkage disequilibrium
MPD: mammalian phenotype database
QTL: quantitative trait loci
SNP: single nucleotide polymorphism
WGS: whole-genome sequence
